# Experiences of family caregivers in green care farms and other nursing home environments for people with dementia: a qualitative study

**DOI:** 10.1186/s12877-019-1163-6

**Published:** 2019-05-28

**Authors:** Bram de Boer, Hilde Verbeek, Sandra M. G. Zwakhalen, Jan P. H. Hamers

**Affiliations:** Faculty of Health, Medicine, and Life Sciences (FHML), Department of Health Services Research (HSR), Maastricht University, CAPHRI Care and Public Health Research Institute, Maastricht, The Netherlands

**Keywords:** Experiences, Green care farms, Informal caregivers, Nursing home choice, Small-scale living

## Abstract

**Background:**

Having a match between a nursing home and the preferences of people with dementia is beneficial for their well-being. It is suggested that innovative types of nursing homes such as small-scale living facilities and green care farms create a better match between their care environment and their residents. However whether this is also reflected into the experiences of informal caregivers is not known. Therefore, this study explores what their positive and negative experiences are with regard to green care farms, other small-scale living facilities, and traditional nursing homes.

**Methods:**

A qualitative exploratory research design was used. Semi-structured interviews with 43 informal caregivers (2014–2015) were carried out. Topics discussed were: positive and negative experiences with the nursing home and reasons for choice of a particular type of nursing home. Thematic analysis including an iterative process of open, axial and selective coding, was used.

**Results:**

Five themes emerged: (1) physical environment and atmosphere, (2) activities, (3) person-centred care, (4) communication, and (5) staff. Informal caregivers at green care farms were more positive about the physical environment, activities, and person-centred care compared with informal caregivers in the other types of nursing homes. Both positive and negative experiences regarding communication and individual staff members appeared across all types of nursing homes.

**Conclusions:**

Experiences of informal caregivers with a nursing home are dependent on the type of nursing home. However, experiences were also often related to individual nursing staff and their interpersonal, ‘human’ qualities.

## Background

Dementia not only influences the lives of the people with dementia themselves, it also has an impact on the lives of the family [1]. Caring for a person with dementia can lead to a significant physical and emotional burden [2]. A substantial group of people with dementia eventually has to be admitted to a nursing home [3]. Resolving on a nursing home admission has been described as the most difficult decision for informal caregivers [4]. In the Netherlands people with dementia and their informal caregivers are free to choose their own nursing home after admission is indicated. There is a wide array of publicly-funded types of nursing homes which people can choose from, including traditional nursing homes, and various small-scale, home-like facilities [5]. Traditional nursing homes often have an institutional character and provide care for a group of 20 or more residents on a ward; nursing staff has differentiated tasks and daily life is mainly determined by routines and rules of the organisation. Small-scale living facilities provide the same care as traditional nursing homes. However, they have a home-like character and provide care for a group of six to eight residents. Nursing staff form a joint household with the residents, meaning they have integrated tasks, and cook, clean, and do other household chores together with the residents [6].

Previous research on the experiences with small-scale, homelike facilities in dementia care showed that family caregivers valued the personal attention that nursing staff provided to resident. The involvement of staff, and the emphasis on autonomy in daily life were appreciated, and family caregivers in small-scale living facilities were more satisfied with the care facility and nursing staff than those in regular (large-scale) nursing home wards [7]. This was supported by other studies showing that group living homes create opportunities for individualized care, attention to residents’ personal needs [8], and reduced levels of informal caregiver burden [9].

Most recently, green care farms providing 24-h nursing home care have been added to the spectrum of available nursing homes for people with dementia. Green care farms are a unique type of small-scale facilities providing nursing home care for people with dementia in a home-like environment on the terrain of a farm [6]. As most green care farms focus on providing day-care services, knowledge of the experiences with green care farm providing 24-h nursing home care is lacking. Up until now, no studies looked into the experiences with green care farms providing 24-h nursing home care. In a study on day care services at green care farms it was suggested people deliberately chose for green care farms because of their dislike of the institutional environment of regular day care facilities. Green care farms were perceived as more useful for clients, more meaningful and providing more opportunities to be physically active and to go outdoors [10]. It is not known whether these findings can be transferred to the nursing home sector. However, a first study suggested that residents at green care farms providing 24-h nursing home have a more active daily life, in which they have more social interactions, and come outdoors more often compared to existing nursing homes [11].

Knowledge on the experiences with different types of nursing homes can be of great importance given the increased focus on person-centred care [12]. An increasing number of nursing homes strives to provide care according to person centred and psychosocial care models. However, this remains a struggle for many nursing homes. Research states that the surroundings of a nursing home should be personalised and that activities should be performed that promote a healthy life as determined by the needs and preferences of the individual with dementia [12, 13]. This indicates that having a match between the needs and wishes of the person with dementia and the environment can promote the delivery of person-centred care [13, 14]. More detailed information about the experiences of informal caregivers with different types of nursing homes can help to gain insight into what nursing homes should focus on when providing person centred care for people with dementia.

## Methods

### Aim

The aim of this study was to explore from the perspective of the informal caregivers of people with dementia, the positive and negative experiences with different types of nursing homes. Experiences with green care farms, regular small-scale living facilities, and traditional nursing homes were explored.

### Design

This study is part of a larger project that studies the impact of green care farms providing nursing home care for people with dementia (*n* = 115) [6]. The current study has a qualitative exploratory research design and investigates what the positive and negative experiences of informal caregivers are with green care farms, regular small-scale living facilities, or traditional nursing homes.

### Setting

Eighteen locations divided over three types of nursing homes were included in this study, all located in the southern part of the Netherlands. Table 1 gives a description of the nursing home types. There is a majority of regular small-scale living facilities because these included both stand-alone facilities and small-scale facilities on the terrain of larger nursing homes. The ratio of staff to residents, and the educational level of staff is the same across settings, and residents have comparable cognitive and functional performance due to a matching procedure in the original study [6, 15]. This matching procedure was carried out 2 week before the original study, meaning that residents participating in the study had comparable cognitive and functional performance at the start of the study.

**Table 1:** Overview of the included types of nursing homes

Nursing home type	Description
Green care farm (5 locations)	Physical environment:
	- A stand- alone small-scale facility
	- Private rooms
	- Common living room/kitchen
	- Combination of private and shared bathroom/shower
	- Familiar furniture/own furniture
	- Access to outdoor areas such as gardens, shed, animal stables, etc.
	Social/organizational environment:
	- Combining care and agricultural activities
	- Approximately eight residents live together
	- Residents and nursing staff form a joint household
	- Steady team of nursing staff with integrated tasks
	- Cooking in the home [6]
Regular small-scale living facility (9 locations)	Physical environment:
	- Small-scale living facilities can be stand-alone in a neighbourhood, or clustered on the area of a larger nursing home.
	- Private rooms
	- Common living room/kitchen
	- Combination of private and shared bathroom/shower
	- Familiar furniture/own furniture
	- Some outdoor spaces available, not always easily accessible
	Social/organizational environment:
	- Daily living is mainly determined by the residents and informal caregivers and approaches a home-like situation as much as possible
	- Approximately eight residents live together
	- Residents and nursing staff form a joint household
	- Steady team of nursing staff with integrated tasks
	- Cooking in the home [5]
Traditional nursing home (4 locations)	Physical environment:
	- Large scale care environment.
	- Combination of private and shared rooms
	- Common living room/kitchen
	- Shared bathroom/shower
	- Often standardized furniture, less familiar
	- Some outdoor spaces available, not always easily accessible
	Social/organizational environment:
	- Approximately 20 residents on a ward
	- Large team of nursing staff with differentiated tasks
	- Daily life is mainly determined by routines and rules of the organisation
	- Cooking in central kitchen, not in the home [5]

### Participants

A convenience sample [16] of the informal caregivers of people with dementia participating in the original research project (*n* = 115) [6] was used in this study. Sampling aimed to include the informal caregiver closest to the person with dementia, who was involved both in the decision making process and in caregiving after admission. Therefore the first contact persons of the resident were asked to participate in the interviews. If they indicated that someone else was closer to the resident, this person was included. As planned in the original proposal [6], participants were invited until no new information emerged from the interviews, following the principle of data saturation. Based on the analyses of all interviews, the research team concluded that data saturation was reached.

### Data collection

Characteristics of the informal caregivers were gathered (i.e. age, gender, marital status, and relationship to the resident). Semi-structured interviews [16] were used. First, the interviews were pilot tested within the research team; the first interview was performed by the first author (PhD, male) and a trained research assistant (MSc, female), who performed all other interviews. There was no prior relationship between the interviewer and the participants before the interviews. Participants received information about the study on paper, and verbally before the interview. The majority of the interviews was conducted at the home of the informal caregiver. The interviews were recorded and, after the interview, a written transcript was made. In order to increase the credibility and confirmability of the data, a member check was conducted: a summary of a subset of the transcripts was given to the participants in order to check whether they agreed on the content of the particular transcript [16].

Three themes were discussed in each interview: 1) informal caregivers’ positive experiences with the nursing home, 2) informal caregivers’ negative experiences with the nursing home, and 3) reasons for the choice of a particular type of nursing home. No specific topics/factors regarding the nursing home were pre-determined, as we wanted respondents to feel free to discuss the topics that would be most important according to their experiences. Table 2 provides an overview of the topic list with example questions. Data collection stopped when themes and categories in the data became repetitive and redundant.

**Table 2:** Topic list and example questions for interview

Topic	Example questions
Positive experiences	“When you look at the nursing home and its direct environment, what do you believe to be positive aspects of the nursing home?”
	“Can you explain why [topic] is important to you?
Negative experiences	“What are experiences you had that in hindsight you see as negative experiences?
	“You mentioned negative experiences about [topic], what do you think are the causes of them?”
Reasons for choice	“What factors did you take into consideration when choosing this nursing home?”
	“Did you have any expectations about the nursing home?”

### Data analysis

**Fig. 1:**
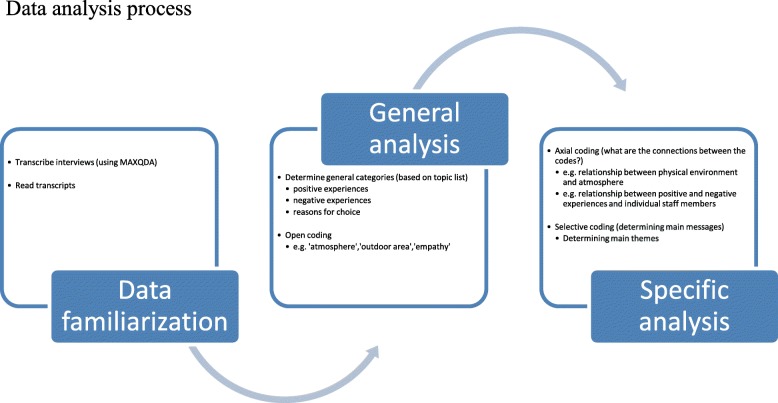
Data analysis process

The data were analysed using qualitative data analysis software MAXQDA [17]. Partly based on a phenomenological research approach aimed at understanding people’s experiences [16, 18], a thematic analyses approach was used, following the steps identified in previous research [19, 20]. The data were systematically searched to identify patterns in order to provide a description of the topics investigated. A combination of open, axial and selective coding was used [16]. First the researchers read through the transcripts several times and started to create labels for chunks of data that summarised the main message. During this stage it was determined whether chunks of data belonged to ‘reasons of choice’, ‘positive experiences’, or ‘negative experiences’, and a label was given to the chunks of data (e.g. ‘reason of choice – close to home’, ‘positive experience – opportunity to participate in activities’, ‘negative experience – lack of communication with informal caregivers’. Relationships between codes were identified by means of axial coding. In this stage of the analysis codes that were identified during open coding were linked. Some themes came to the front as being important factors in the choice of a particular nursing home, and were also often mentioned when talking about positive and negative experiences (e.g. ‘activities’, ‘communication’, ‘physical environment’). Keeping these main points of interest in mind, selective coding led to the core themes discussed in the current paper. During this last stage, nuances, and differences between the types of nursing homes were identified. Figure 1 shows an overview of the steps taken during the analyses. The whole analysis was an iterative process. Indicating that for instance during open coding, sometimes relationships could already be identified, or that during axial coding, main themes already emerged. The interviews were coded by the first author and independently checked by the second author throughout the analysis process. In case there was disagreement, decisions were made based on discussion within the whole research team.

## Results

In total, 85 informal caregivers were approached for an interview, 43 informal caregivers were interviewed (14 informal caregivers declined to participate; the rest were not available at the time of the data collection). The interviews lasted on average 37 min, with a range of 12–76 min. 15 participants received a summary of the manuscript, all of them agreed with the content of the transcripts during the member check. Table 3 gives an overview of the sample characteristics of the informal caregivers and the people with dementia.

**Table 3:** Characteristics informal caregivers and nursing home residents with dementia

	Total (*N* = 43)		Traditional nursing home ward (*N* = 11)		Green care Farm (*N* = 10)		Regular small-scale living facility (*N* = 22)^a^	
Informal caregiver								
Age (SD)	58 (11)		64 (13)		54 (10)		56 (8)	
Gender	F	M	F	M	F	M	F	M
	25	18	6	5	5	5	14	8
Relationship (% son/daughter)	74%		45%		70%		96%	
Marital status informal caregiver (% married)^b^	79%		80%		88%		75%	
Persons with dementia								
Age (SD)	84 (8)		84(10)		81(9)		85(6)	
Gender^c^	F	M	F	M	F	M	F	M
	35	8	7	4	7	3	21	1
S-MMSE (SD)^d^	8 (6)		6 (7)		7 (6)		10 (5)	

^a^Regular small-scale living facilities consist of two types: stand-alone living facilities in a neighbourhood and small-scale living facilities on the terrain of a larger nursing home

^b^Five participants did not share their marital status

^c^Significant difference at α = .05

^d^S-MMSE = Standardized Mini-Mental State Examination is used to assess cognitive impairment. Scores range from 0 to 30, with higher scores indicating better cognition

### Experiences with the care environment

The positive and negative experiences of informal caregivers with the care environment could be clustered within five themes: (1) physical environment and atmosphere, (2) activities, (3) person-centred care, (4) communication, and (5) staff. In general, informal caregivers agreed upon what they evaluated as positive and negative regarding these themes. For the first three themes, having solely positive (or negative) experiences was related to the type of nursing home.

### The physical environment and atmosphere

Differences between the types of nursing homes were found in experiences with the physical environment and the atmosphere. Informal caregivers of residents in green care farms and regular small-scale living facilities had more positive experiences compared with informal caregivers of residents in traditional nursing homes. They valued the opportunities that the physical environment provided for encouraging residents. Furthermore, the familiar home-like environment was appreciated.

‘I really think the small-scale environment is important. It just has a certain look and feel to it. I remember that when I first came here, the only thing reminding me of the fact that this was a ‘care facility’ was the chair in the shower. But everything else is like entering an ordinary house. So actually this is kind of a ‘home’.’ [green care farm 1620]

Avoidance of traditional nursing homes with a clinical atmosphere was mentioned as a reason to choose a small-scale, home-like facility or a green care farm. Informal caregivers elaborated on this, stating that they did not want their relative to live in facilities that they often described as ‘hospital-like settings’.

‘Well, we had a very clear image of what we didn’t want. We did not want a large facility in an apartment building with long corridors and large groups of people living together with changing nursing staff. Food coming fully prepared from a general kitchen and activities only being performed in large groups. That is what we didn’t want. So then you start looking for small-scale facilities.’ [green care farm1601]

Regarding traditional nursing homes, informal caregivers mentioned that a home-like feeling was missing. A clinical atmosphere was mentioned as a negative experience of the care environment.

‘The ward doesn’t look like a home at all. They have a common living room, which looks like a waiting room in a hospital. They have these large plastic chairs, which doesn’t create a home-like feeling.’[traditional nursing home 110]

### Activities

All informal caregivers mentioned that activities were important for nursing home residents. However the focus differed across settings. At regular small-scale living facilities and in traditional nursing homes, informal caregivers mentioned that there were many centrally-organised activities such as games (bingo), music, or other organized activities. People thus often opted for a traditional nursing home because they provide residents with a lot of centrally organised activities for entertainment.

‘Last week they went to the zoo, which was great! And Sunday they had a barbeque, also very nice. And they have something to do almost every day; one day they have bingo, and the next day they have music.’[traditional nursing home 1004]

At green care farms informal caregivers were very positive about the amount of activities, and the autonomy residents have with regard to doing activities.

‘The fact that people have the freedom. They have a large garden with all sorts of chickens, cows, and goats. And if they want they can go to them. They are occupied in the gardens, with growing vegetables and stuff. People can just do things on their own, without having the feeling they have to ask for permission first. They are free to walk around.’[green care farm 501]

Furthermore, the way meaningful activities were integrated into daily life was appreciated. Informal caregivers mentioned several concrete examples such as the fact that residents had the opportunity to participate in farm-related activities such as gardening, and feeding the animals, but also in domestic activities such as doing the dishes or cleaning.

Looking at negative experiences regarding activities, in contrast to green care farms, informal caregivers at traditional nursing homes and regular small-scale living facilities mentioned that residents were still passive for a large proportion of the day, which they perceived as negative. Residents spend a lot of time just sitting in a chair while nothing happens. Furthermore, examples were given of the lack of stimulation to be active, and the fact that activities were organised in an inappropriate way.

‘When she wants to get up to clean the table, they tell her to remain seated, and that they [staff] will do it. Whereas, on other wards, I’ve seen that they ask residents to set and clean the table, to do the dishes, and that kind of stuff.’ [regular small-scale living facility 301]

‘They [staff] take over everything. In the morning they wash and dress him. Then he goes to have breakfast and they ask him what kind of sandwich he wants. So he doesn’t have to make it himself. Then he goes to his room and just sits there. And around 12 he gets a warm meal which is already made for him. So there is nothing that stimulates him.’ [traditional nursing home 1015]

In general, across all types of nursing homes, activities were mentioned as an important factor. The fact that people with dementia could continue the lives they had before admission as far as possible was very important in green care farms and regular small-scale living facilities. People chose small-scale living facilities (including green care farms) because of the integration of activities in routines of everyday life. Activities were perceived as meaningful and giving residents the opportunity to contribute something.

‘So we also looked at what she could do here, and she can pick up the eggs here, she can get milk from the cows. And if she wants she can work in the garden, or just have a walk outside. There are goats, chickens, rabbits, cows, and the way life is, it is just like a real farm, that’s it.’ [green care farm 408]

### Person-centred care

In general, informal caregivers highly valued experiences of the nursing staff as kind, empathetic, patient, paying personal attention to residents, and taking wishes and preferences of residents into consideration while providing care. Practical examples included when nursing staff took into consideration the way they addressed residents, and when they payed attention to residents’ preferences during dinner.

‘They approach her the way she wants to be approached. So all that formal stuff like addressing residents by their surname isn’t like her. So they address her with her first name.’ [regular small-scale living facility301]

‘What I really appreciate is that they address people with their last name. Sometimes you hear them using first names, but my mother would not be amused when they would do that to her.’ [regular small-scale living facility 801]

Differences were found across settings. Informal caregivers at green care farms experienced a higher level of person-centeredness compared with traditional nursing homes and regular small-scale living facilities, where, by contrast, informal caregivers mentioned that staff had a lack of time, and were too busy.

‘You get the feeling they really have time for the residents; they are not in a hurry because they need to do other stuff. Probably, in the background they do, but we don’t notice it. They just really pay personal attention to people.’ [green care farm 1601]

‘They need to pay more attention. Just go to people. Sit with them at the bed when you [staff] have a quiet moment, instead of sitting behind the computer. Just sit with them and get in contact with the people. Just have a chat, and I’m not saying nobody does this, because I noticed it is also related to the person who is working.’ [regular small-scale living facility 1405]

When talking about why choosing a particular setting, person centred care was also mentioned as an important factor. At green care farms, informal caregivers had actively looked for a nursing home that matched the preferences, backgrounds, and living experiences of their relatives.

‘We chose this care farm because my father has an agricultural background. He grew up on a farm and is familiar with the life of a farmer. He was a sheep breeder, which has been his most important occupation. And because this is a farm where there are a lot of animals and vegetables being cultivated it is like going back to his roots.’[green care farm 506]

The characteristics of small-scale, living facilities, in which a situation as closest to home is stimulated, was mentioned as an important factor by informal caregivers in choosing this type of nursing home.

‘This small-scale living facility is just more home-like (than a traditional nursing home). There are always the same nursing staff, which makes it easier to get to know each other. Furthermore, the residents can also help with cooking, for instance by peeling the potatoes. These things they have done all their lives, and here they can still do this.’ [regular small-scale living facility 1102]

### Communication

The importance of communication between all people involved (informal caregivers, nursing staff, management) was a topic that was mentioned consistently by informal caregivers as influencing their experience. Both positive and negative experiences were mentioned in all three types of nursing homes.

‘Yes! I feel it is important that they [staff] can share anything they want with the family. And I particularly think it is important that we feel we [the family] can share what we think is important for our mother. I think that’s the most important thing, if you don’t have that kind of contact with each other, then you can’t trust each other.’ [regular small-scale living facility 202]

Participants agreed that good communication was important for gaining trust and getting to know each other, being beneficial for both nursing staff and family of residents. Open, transparent communication was appreciated, as informal caregivers liked to stay up to date on the experiences of their relative.

‘Every week our whole family receives a short summary from the manager on what happened during that week. About things that went well, and things that did not go well. And of course, it is about positive and negative things, but I appreciate receiving this information. And we can also reply to these messages, and if I come here and I haven’t read the last message. I can just look into the file of my mother.’[green care farm 408]

Negative experiences with communication had to do with the fact that there was no common communication strategy among nursing staff. Informal caregivers indicated that there are large differences between nursing staff regarding the information they communicate to the family. Some staff were said to really elaborate on even the smallest details, whereas other failed to share major events.

‘Well, it depends on the individual nursing staff. Not everyone is involved with the people in the same way. One mentions immediately when my mother has issues with something whereas the other doesn’t say anything. With some I have to accidently heard them talking about it and really ask questions about it, otherwise they don’t share anything with me.’[regular small-scale living facility 704]

Another aspect that was considered negative was poor communication between nursing staff. Informal caregivers mentioned that they repeatedly have to ask multiple nursing staff the same questions, whereas nursing staff should communicate with each other in order to increase uniformity in the care that is being provided. Otherwise, this can lead to adverse effects.

‘At the beginning they said that my mother would remain in bed for one day. But the nurse from today does not know she was in bed the entire day yesterday, and the day before that. So at certain times she stayed in bed for three days a week. Even until 3 o’clock in the afternoon, or until supper. And that is not how it should be. [regular small-scale living facility 801]

### Staff

Both positive and negative experiences could often be attributed to individual staff members rather than to the type of nursing home. Therefore, ‘staff’ was identified as a separate theme. The importance of the staff’s role was emphasised in all interviews. Positive and negative experiences regarding communication, activities, and person-centred care appeared related to individual staff members. Informal caregivers mentioned that the way nursing staff were able to provide good care had to with what they ‘brought to the table’ as a human being, instead of a professional. Aspects such as empathy, compassion, and authenticity were felt to be important and some staff members were said to possess these qualities more than others.

‘It depends; some nursing staff chooses to do something with the residents when they have a quiet moment, whereas others don’t. I actually heard one nurse say “I’m not trained to occupy the residents, I’m here to care for them.”’ [regular small-scale living facility 704]

‘Some nurses completely ignore us when we are here; they hardly say anything to us. But this varies a lot between nurses. Some nurses have more feeling with this than others.’ [regular small-scale living facility 607]

## Discussion

This study explored the positive and negative experiences of informal caregivers of people with dementia with different types of nursing homes. Experiences with green care farms, regular small-scale living facilities, and traditional nursing homes were explored. Positive and negative experiences were found within five themes: (1) physical environment and atmosphere, (2) activities, (3) person-centred care, (4) communication, and (5) staff. Informal caregivers at green care farms were more positive about the physical environment, activities, and person-centred care compared with informal caregivers in the other types of nursing homes. Both positive and negative experiences regarding communication turned up across all types of nursing homes. Experiences were often dependent of the relationship and role of individual staff members taking care of the resident, irrespective of the type of nursing home.

The finding that communication was identified as an important theme is in line with previous studies. The role of communication in nursing home care is well established [21, 22]. Especially for people with dementia, it is important to focus on aspects such as mutuality, autonomy, respect, and trust during communication [23]. This study showed that the capacities for especially traditional nursing homes to provide residents with person-centred care in a home-like, familiar atmosphere where residents are stimulated to be active remained a problem. This is in line with previous studies showing that residents of traditional nursing homes spend the majority of their time doing little or nothing, without having social interaction [11, 24, 25]. Furthermore, previous studies also found that informal caregivers of residents in small-scale living facilities expressed positive experiences of their contact with nursing staff, personal attention, and the autonomy of residents at small-scale living facilities [7, 26].

A cultural change towards more person-centred care in nursing homes is occurring [27–29]. More research is needed on how we can implement successful elements of green care farms and other types of nursing homes, such as taking the preferences and remaining capacities of people with dementia as a starting point, and providing a stimulating environment into traditional nursing homes [29–31].

Lastly, this study showed that it is sometimes difficult to determine whether positive and negative experiences should be attributed to the type of nursing home, or to individual staff members. Aspects such as empathy, compassion, and authenticity were felt to be important and some staff members were said to possess these qualities more than others. Previous studies suggest that the educational level and competencies of nursing staff play an important role with regard to the quality of care at a nursing home [32]. Considering the increased focus on person-centred care skills such as active listening, emotional recognition, and empathetic ability are becoming increasingly important [33]. Therefore, more focus is needed, in both research and practice on how to improve the competencies and abilities of nursing staff. The leadership of managers, or other role models in a nursing home, might play a key role in this as they are needed to coordinate, coach and evaluate the skills of nursing staff [34] to optimise care provided. It is important, that next to professional competences, future education for nursing staff focuses on these ‘human qualities’ as well.

Some methodological considerations have to be made. As we used a convenience sample, selection bias might have occurred. It is possible that informal caregivers whose experiences were mainly positive were more willing to take part in the study. Furthermore, it is possible that characteristics of the participants, and the people with dementia might have influenced the reported experiences. A matching procedure was carried out at the start of the original study (on the total sample of residents). However, as the current study interviewed informal caregivers of a subset of the total sample, it is possible some differences between facilities emerged. For instance, on the informal caregiver level, differences in terms of the relationship (son/daughter, spouse, etc.) exist. In addition, it is not clear whether characteristics at the time of admission differed, as we did not gather this information. We cannot rule out the possibility that these characteristics influence the experiences with a particular type of nursing home. The current study did not include the perspective of the people with dementia, whereas their experiences and opinions can lead to relevant information for practice. Future studies should incorporate the views of people with dementia, and should focus on using purposive sampling techniques. In addition, making use of explicit memo’s or field notes during data collection to enhance researcher reflexivity is recommended.

## Conclusions

This study showed that the experiences of informal caregivers with a nursing home are dependent on both the type of nursing home, as well as individual nursing staff. Informal caregivers perceived green care farms as better able to provide residents with a stimulating environment that provides person-centred care, compared with traditional nursing homes. However, experiences were also often related less to the professional competencies of individual nursing staff than to their interpersonal, ‘human’ qualities.
